# Supplementary material for: Cross-species genomic landscape comparison of human mucosal melanoma with canine oral and equine melanoma
Source: Nat Commun. 2019 Jan 21;10:353. doi: 10.1038/s41467-018-08081-1 (PMC6341101; doi:10.1038/s41467-018-08081-1)
Supplement: Supplementary file 1 — Supplementary Information [file 41467_2018_8081_MOESM1_ESM.pdf]

Cross-species genomic landscape comparison of human mucosal melanoma with canine oral and equine melanoma

Supplementary Information

Wong *et al*.

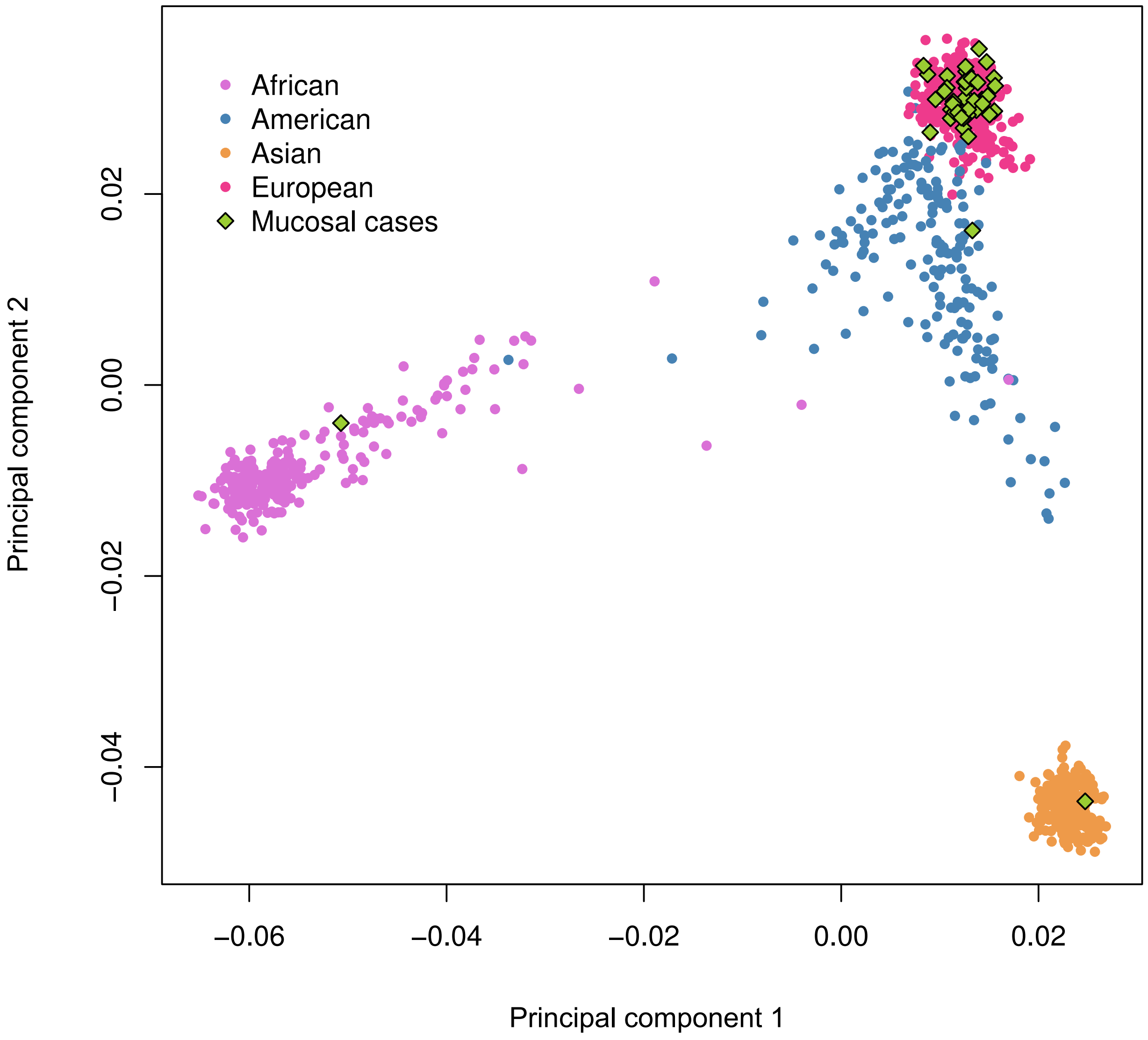

Supplementary Figure 1: Principal component analysis plot depicting the ancestry of 46 germline samples from patients with mucosal melanoma. The plot shows the first and second principal components. Ancestry was estimated using the 46 mucosal germline samples and the 1000 Genomes Project Phase I samples (1,092 individuals) together.

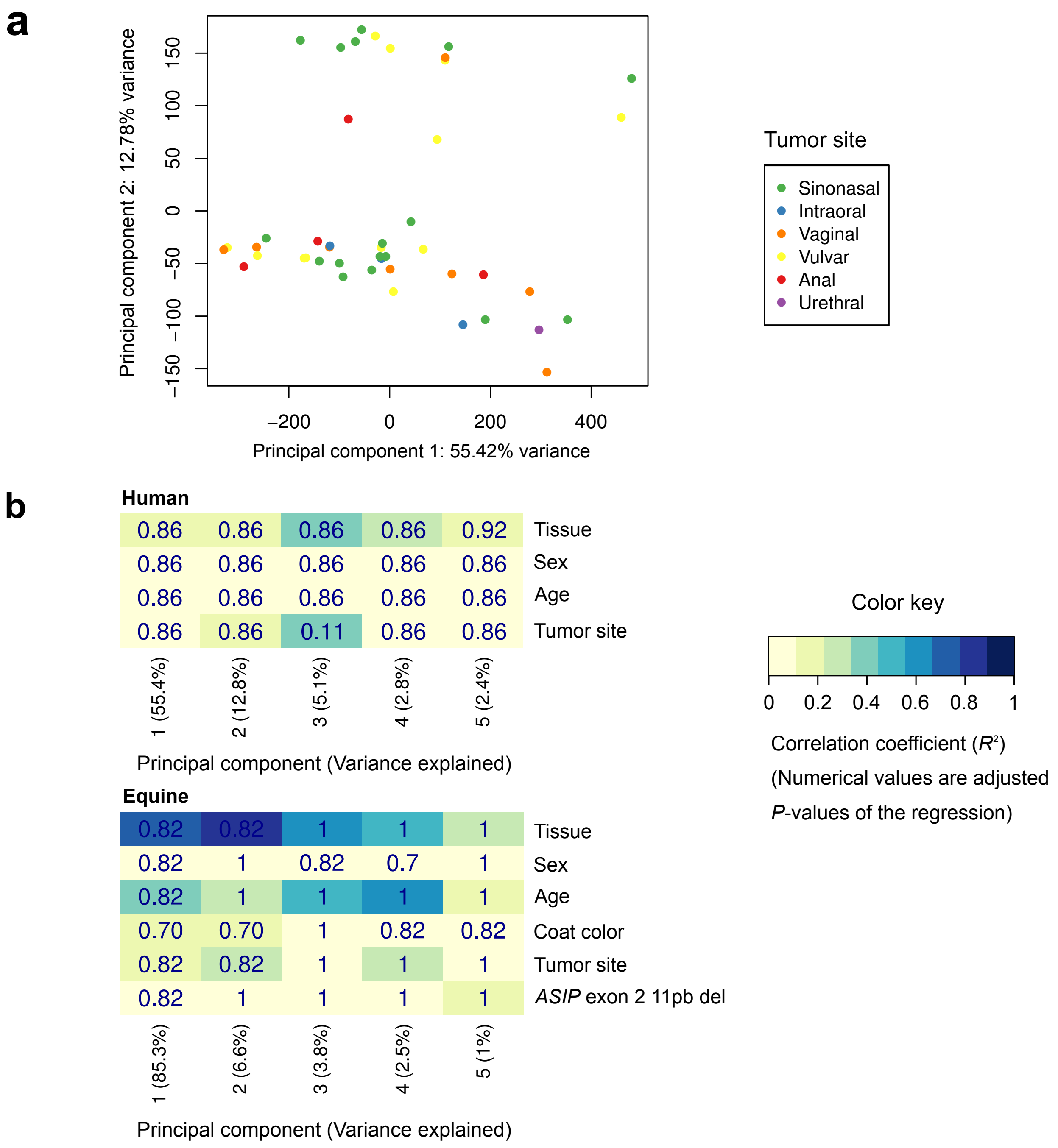

Supplementary Figure 2: Principal component analysis of mutation data from human mucosal and equine melanoma samples sourced from various tissue sites. (a) Principal component analysis plot of human mutation data de-sparsified into biological data pathways. (b) Heatmap of the correlation coefficients (R^2), as indicated by the color key, from principal component (PC) regression against phenotypic variables in the human and horse datasets. Numbers inside of the heatmap cells are the adjusted *P*-values of the regression. The first five PCs are listed on the *x*-axis, and the percentage of variance that is explained by each PC is given inside parentheses. Phenotypic data are derived from Supplementary Data 1. Samples from specific tissues were grouped into tumor sites, which are shown in Figures 1 and 2 and listed Supplementary Data 1. There was no significant correlation between the first 5 PCs and any of these variables.

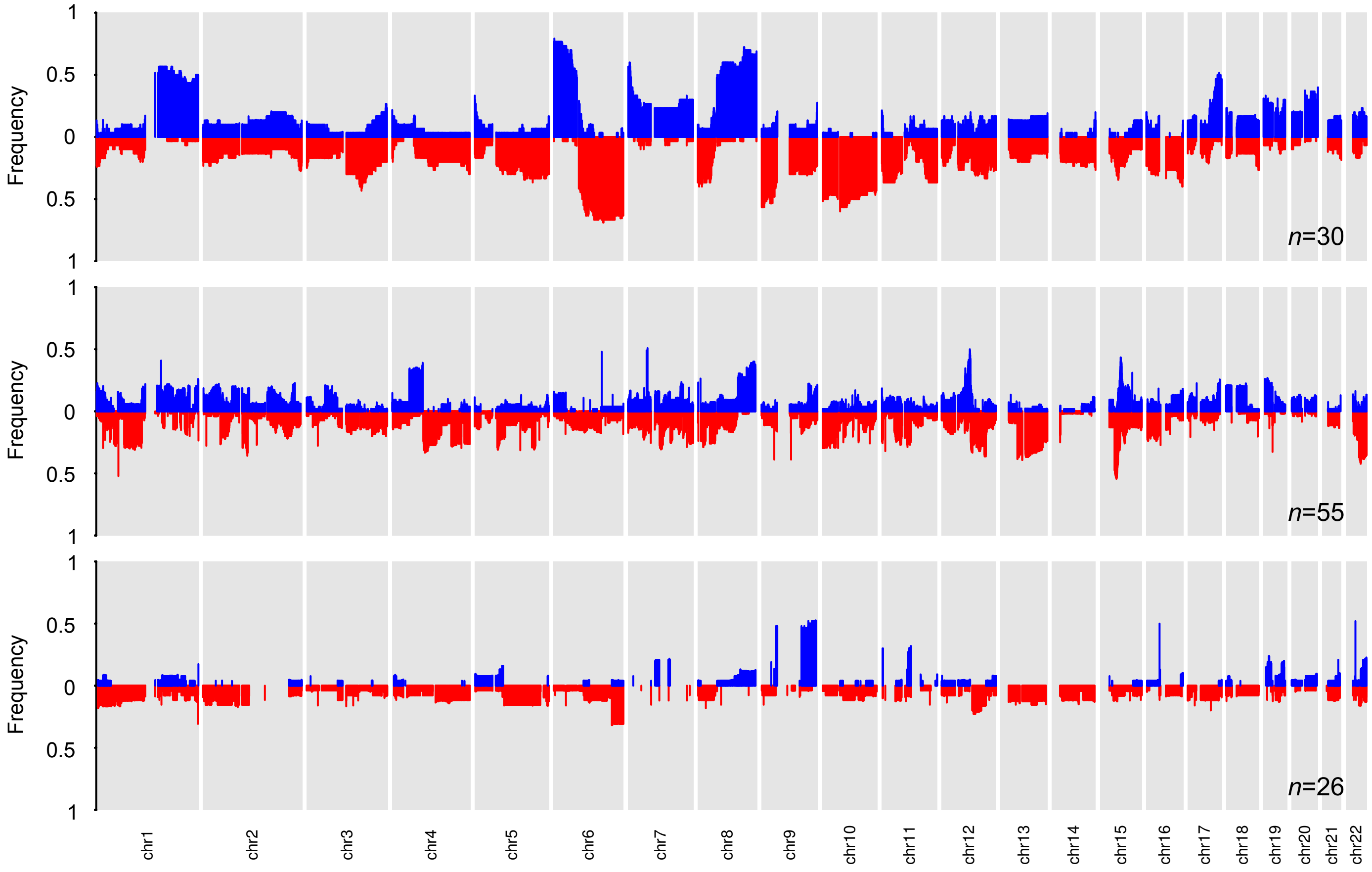

Supplementary Figure 3: Comparison of somatic copy number alterations in primary human, canine and equine mucosal melanoma by mapping of canine and equine sequences to orthologous regions on the human genome. To generate copy number (CN) frequency profiles for visual comparison, we used a method similar to one described previously (see Methods) to map CN frequencies within canine and equine regions to orthologous regions on the human genome (GRCh38). We first used the liftOver tool (see Methods) to map genomic regions corresponding to the Agilent SurePrint G3 Human CGH 4x180K Microarray 60mer probes (which are provided relative to hg18) to GRCh38. The CN frequencies (blue, CN gain; red, CN loss) within the human melanoma cohort were plotted for each of the re-mapped regions (top). Each 60mer region was then mapped to CanFam3.1 to determine the CN frequency of the orthologous region in the canine melanoma cohort, and the CN frequecies were plotted relative to GRCh38 (middle). The procedure was repeated for the equine melanoma cohort by mapping the 60mer regions to EquCab2.0 and plotting CN frequencies relative to GRCh38 (bottom). See Methods for additional details. Several recurrent CN alterations shared between species can be visualized, including a focal deletion on chromosome 15 (human and canine), amplification of the distal end of chromosome 8 (human and canine), partial deletion of chromosome 12 (human, canine and equine), and deletion of the distal end of chromosome 6 (human and equine).

	Human	Canine	Equine
**Signatures in cohorts**			
Samples	59	71	62
Total mutations	5559	2238	17238
Signature (*cosine* similarity)	7 (0.96*)<br>1 (0.80*)<br>5/30 (0.74/0.75)	1 (0.87*)	7 (0.96*, mucosal)<br>7 (0.94*, all other)
**Signatures in samples (min. 100 mutations)**			
Samples	11	1	5
Total mutations	3023	N/A	16715
Signature (*cosine* similarity)	7 (0.88*)<br>5 (0.79)<br>1 (0.68)	N/A	7 (0.96*, mucosal)<br>7 (0.94* all other)
Samples with UV signature (7)	PD25643a (nasal mucosa)<br>PD25643c (nasal mucosa)<br>PD25657a (nasal mucosa)<br>PD26932a (lip, mucosal surface)		HD0021a (mucosal)<br>HD0032a (mucosal)<br>HD0071a (cutaneous)<br>HD0083a (cutaneous)<br>HD0084a (cutaneous)

Supplementary Table 1: Mutation signatures in human mucosal, canine oral and equine melanomas. Mutation signature decomposition was performed on tumor samples from each species cohort, including recurrences and metastases where available. Signatures were identified in whole cohorts and in specific samples with a minimum of 100 mutations. The c*osine* similarity was used to compare the extracted signatures to the 30 COSMIC signatures from Alexandrov *et al*.[1] (see Methods). Signatures 1 and 5 are seen in all cancer types. Signature 1 is associated with age and 5-methylcytosine deamination, while the aetiology of signature 5 is unknown. Signature 7 is associated with UV light exposure. Samples in which signature 7 comprised at least 50% of the total signature contribution are shown. *Match to the COSMIC signature is confirmed based on high *cosine* similarity and visual inspection of the signature profiles.

Supplementary Reference

[1]Alexandrov, L. B. *et al*. Clock-like mutational processes in human somatic cells. *Nat*. *Genet*. 47, 1402-1407, doi:10.1038/ng.3441 (2015).
