# Supplementary material for: Cross-species genomic landscape comparison of human mucosal melanoma with canine oral and equine melanoma
Source: Nat Commun. 2019 Jan 21;10:353. doi: 10.1038/s41467-018-08081-1 (PMC6341101; doi:10.1038/s41467-018-08081-1)
Supplement: Supplementary file 3 — Description of Additional Supplementary Files [file 41467_2018_8081_MOESM3_ESM.docx]

**Description of Additional Supplementary Files**

**Supplementary Data 1: Clinical details for the human, canine and equine samples sequenced in this study**. Each primary tumor sample is grouped with its matched normal sample, and metastases and/or recurrences from the same patient, if available. If a metastatic or recurrent tumor was compared to a different matched normal sample than the primary tumor (*e.g.*: cores were taken from adjacent normal tissue for both the primary and metastasis), the normal sample is listed below it. “Tissue/site” is the location from which the each sample was obtained. These are the original annotations from pathologists. Primary tumors were grouped into broader categories by site (“Primary tumor site”). All tumors from the (sino)nasal cavity/sinus were from the nasal mucosa. The categories in the “Primary tumor site” column were used to group primary tumors for Figures 1 and 2. The human metastases to lymph nodes were all locoregional metastases. Recurrences were all locoregional. For each equine germline sample, exon 2 of the *ASIP* gene was examined for a polymorphic 11 bp deletion which is associated with increased incidences of melanoma in grey horses. WT: wild type (no deletion); Het: heterozygous for the deletion; Hom: homozygous for the deletion.

**Supplementary Data 2: Somatic mutations in human mucosal, canine oral and equine melanoma.** Shown are SNVs, MNVs and indels identified in coding regions and splice sites, in canonical transcripts as defined in Ensembl v89 (human) and Ensembl v91 (canine and equine). Listed are somatic mutations in 46 primary, 5 recurrent, and 8 metastatic human mucosal melanomas; 65 primary and 6 metastatic canine oral melanomas; 28 primary equine melanomas from mucosal-like and mucocutaneous sites; and 34 primary equine melanomas from cutaneous and other non-mucosal-like and non-mucocutaneous sites. No SNVs or indels were found in equine samples HD0024a and HD0076a. Coordinates are relative to the GRCh38 (human), CanFam3.1 (canine) and EquCab2.0 (equine) reference genomes.

**Supplementary Data 3: Orthology relationships between human, canine and equine genes.** Listed are orthology relationships between human and dog, and human and horse, as predicted by Ensembl Compara for Ensembl release v91, for Figures 1, 2 and 5. Canine *MAP2K2* has been assigned as orthologs to human ENSG00000126934 (*MAP2K2*) and ENSG00000230626; however the similarity score (96.75%), genome order conservation (GOC) score and whole genome alignment coverage score all higher with ENSG00000126934 (*MAP2K2*). The orthology relationship of equine *DNAH5* is shown relative to canine *DNAH5*; no relationship is listed for human and dog or human and equine *DNAH5* in Ensembl v91. Canine and equine genes that have not been assigned a gene symbol or ortholog were not included in Figures 1, 2 or 5.

**Supplementary Data 4: Cross-species comparison of large recurrent DNA copy number aberrations.** Syntenic regions with recurrent somatic copy number gain or loss across chromosome arms or whole chromosomes. Coordinates are relative to the reference genomes GRCh38 (human), CanFam3.1 (canine) and EquCab2.0 (equine). Syntenic regions were obtained from the Ensembl Compara database. The minimum copy number frequency required for interspecies comparison was 0.20. For each chromosome arm (human) or chromosome (canine and equine), the median frequency of copy number gain (or loss) was calculated from 1Mb windows. Samples included in this analysis are also listed. As described in the Methods, some samples with excessive noise were excluded from somatic copy number calling with Sequenza, and subsequent downstream analysis with GISTIC 2.0 and/or STAC.

**Supplementary Data 5: Cancer Gene Census genes located in significantly amplified and deleted regions in human mucosal melanoma**. Shown are peaks from GISTIC 2.0 “region limits” and “wide peaks”, and genes within these regions that are found in the Cancer Gene Census catalog. Definitions from GISTIC 2.0 are as follows: Region Limits: boundaries of the entire significant region of amplification or deletion; Wide Peak Limits: most likely to contain the targeted genes; *q*-values: the *q*-value of the peak region; Residual *q*-values: the *q*-value of the peak region after removing (“peeling off”) amplifications or deletions that overlap other, more significant peak regions in the same chromosome. Green: gene is within wide peak limits only; Blue: gene is within region limits only; Black: gene is within both wide peak and region limits. Shown are all results with residual *q*-value < 0.2. Regions in red had *q*-values > 0.05 and were not considered significant.

**Supplementary Data 6: Significant DNA copy number aberrations from STAC analysis.** Listed are copy number aberrations in human mucosal melanoma, canine oral melanoma, and equine melanoma from mucosal-like and mucocutaneous sites. Copy number amplification or deletion frequencies in 1Mb regions (STAC "locations") were used for analysis by STAC. Locations with a frequency *P*-value < 0.05 and/or a footprint *P*-value <0.05 and a location frequency of at least 0.2 were considered significant (see Methods). Cancer Gene Census genes with whole or partial overlap with each location are shown. Adjacent significant locations were merged to determine region boundaries. Regions less than half a chromosome arm (human) or chromosome (canine and equine) in size were used for comparison of focal aberrations.
